# Examining Safety of Biocolourants from Fungal and Plant Sources-Examples from *Cortinarius* and *Tapinella, Salix* and *Tanacetum* spp. and Dyed Woollen Fabrics

**DOI:** 10.3390/antibiotics9050266

**Published:** 2020-05-20

**Authors:** Riikka Räisänen, Anja Primetta, Sari Nikunen, Ulla Honkalampi, Heli Nygren, Juha-Matti Pihlava, Ina Vanden Berghe, Atte von Wright

**Affiliations:** 1 Craft Studies, University of Helsinki, P.O. Box 8, 00014 Helsinki, Finland; anja.primetta@helsinki.fi (A.P.); sari.nikunen@gmail.com (S.N.); 2 Department of Environmental and Biological Sciences, University of Eastern Finland, P.O. Box 1627, 70211 Kuopio, Finland; 3 Department of Pharmacology and Toxicology, University of Eastern Finland, P.O. Box 1627, 70211 Kuopio, Finland; u.honkalampi@innolact.fi (U.H.); atte.vonwright@uef.fi (A.v.W.); 4 VTT Technical Research Centre of Finland Ltd., P.O. Box 1000, 02044 Espoo, Finland; heli.nygren@vtt.fi; 5 Natural Resources Institute Finland (Luke), Tietotie 4, 31600 Jokioinen, Finland; juha-matti.pihlava@luke.fi; 6 Royal Institute for Cultural Heritage (IRPA/KIK), Parc du Cinquantenaire 1, 1000 Brussels, Belgium; ina.vandenberghe@kikirpa.be

**Keywords:** natural dye, biocolourant, secondary metabolite, in vitro, cytotoxicity, mouse hepatoma cell, highest tolerated dose, HPLC-UV/Vis-MS

## Abstract

Biocolourants have been investigated as alternatives to synthetic dyes. However, natural origin per se is not a label of harmlessness and research is needed to obtain safe dyes. We studied the cytotoxicity of the extracts from fungal (*Cortinarius semisanguineus*, *Tapinella atrotomentosa*) and plant (*Tanacetum vulgare, Salix phylicifolia*) sources and the woollen fabrics dyed with the extracts. Cytotoxicity in vitro using hepa-1 mouse hepatoma cells for 24 h and 72 h exposure was observed as the highest tolerated dose. All biocolourants produced intensive colour on fabrics with fastness properties from moderate to good. The *Salix* and *Cortinarius* samples did not show any cytotoxic effects, whereas the *Tanacetum* and *Tapinella* samples had slightly higher test values but were not interpreted as being significantly toxic. Higher than zero values of the undyed fabrics showed the importance of examining their toxicity as well. It was found that the cytotoxicity of the samples dyed with the biocolourants did not differ significantly from the undyed wool fabric. The concentrations of dyes used in the assays were very low, imitating the dose of the user. In addition to colouring properties, natural dyes may have pharmaceutical and antibacterial properties which would enhance the interest in using them in products for added value.

## 1. Introduction

### 1.1. Background

The environmental disadvantages of synthetic dyes are well known. The textile industry discharges large amounts of highly coloured effluent wastewater, the dyes of which severely affect photosynthesis and aquatic life. Effluents may also contain toxic amounts of metal ions and chlorine [1]. In textile workers and end-users, synthetic colourants may cause adverse reactions, such as allergies, urticaria and dermatitis, or respiratory difficulties like asthma, in addition to cytotoxic, genotoxic, carcinogenic and mutagenic effects [2,3,4,5].

Alternative colourants among secondary metabolites from plant and microbial sources such as fungi, yeast and algae have been explored. The natural origin does not automatically mean non-toxicity. Also, the different stages of the textile production process have an impact on the final product, its safety to the consumer, and the environmental load. The degradability of biocolourants is a positive feature in circular material production, whereas in the textile dyeing process, the requirements of colourants are set in high affinity to fibres and stability under end-use conditions. Thus, the utilisation of bio-based colourants requires careful consideration of dye technical and toxicological properties as well as economic feasibility, in which the multi-functionality through antimicrobial and UV-protective properties can create interesting prospects.

Our aim in this article is to examine the information about the safety of natural colourants and the results from the preliminary tests of a few dyes of fungal and plant origins. In this study, fungi surprise webcap [*Cortinarius semisanguineus* (Fr.) Gillet] and velvet roll-rim [*Tapinella atrotomentosa* (Batsch.) Šutara] and plants tansy (*Tanacetum vulgare* L.) and willow bark (*Salix phylicifolia* L.) were used as sources of biocolourants for dyeing of woollen fabrics. 

### 1.2. Safety and Medicinal Use Related to Fungal and Plant Colourants

Correct identification and characterisation of the biocolourant’s source is crucial when assessing potential toxicity, as toxic compounds are concentrated in certain taxa [6,7]. Different taxa, whether fungi or plants, also have their own typical colourants [8,9,10,11]. For *Rubia* and *Cortinarius* genera, the anthraquinones provide a variety of red hues, whereas yellow-orange flavonoids and carotenoids can be obtained from *Tanacetum* species, and bluish-violet terphenyl quinones from *Tapinella* fungi [12,13].

Plant parts such as stems, leaves, flowers, roots, bark, berries, and cones vary in the composition and contents of biocolourants according to the developmental stage and harvesting time [6,10,14,15,16,17]. The contents may also vary depending on the geographical area, cultivation practices and storage conditions [18,19,20,21,22].

The pre-handling steps such as drying and grinding and subsequent extraction conditions (e.g., solvent) affect the content of biocolourants [13,23,24,25]. However, after drying at 45 °C for 1 week, milling to a powder and storage for more than a year, at least two native enzymes in madder roots (*Rubia tinctorum* L.) were still highly active upon addition of water, and depending on the conditions, caused conversion of molecules into forms, some of which were toxic [26].

Natural dyes are complex mixtures of dye components, setting challenges for industrial utilisation. Repeatable colour can be achieved by using standardised extraction and quality analyses of the dye components in the mixtures or by producing single component purified dyes [13,25]. The improved repeatability of colour components will also minimise variation of other secondary metabolites and risks of toxic properties. 

By understanding reaction mechanisms and how the reactions can be controlled [26], the safety risks can be minimised. Those reactions may also affect the dyeing potential, as the products of hydrolysis, i.e., aglycone structures, differ in their properties and affinities to various materials compared to their derivatives [9,13]. The historical background does not necessarily give any guarantee of safeness as even widely-used dye sources can contain toxic secondary metabolites: madder root extracts (*R. tinctorum*) having alizarin (**1**) as the main anthraquinone aglycone, also contained toxic lucidin (**2**), rubiadin (**3**) and xanthopurpurin (**4**) (Table 1) [26,27,28,29,30], of which lucidin and xanthopurpurin were hydrolysis and decarboxylation products of lucidin primeveroside and munjistin. Furthermore, it has been proposed that lucidin is converted into other compounds, such as nordamnacanthal or quinone methide [26,28,31] or degrade to xanthopurpurin.

Toxicity studies of colour providing secondary metabolites, e.g., those of anthraquinones, have given somewhat contradictory results. Jäger et al. [27] detected lucidin (**2**), rubiadin (**3**) and xanthopurpurin (**4**) being mutagenic, but not alizarin (**1**) (Table 1), in madder root extracts (EtOAc). However, all the madder dyed wool samples had mutagenic effects in Ames assays (strain TA100). Previously, Yasui and Takeda [29] had found that the madder root extracts (EtOAc) were mutagenic for both strains of *S. typhimurium* (TA100 and TA98). They concluded that all mutagenic activity found in the EtOAc extracts was due to lucidin. However, Kawasaki et al. [32] suggested that the mutagenicity of the extracts of *R. tinctorum* was not exclusively due to lucidin but also a result and contribution of other mutagenic compounds.

The chemical structures of secondary metabolites and their functional groups, for example, hydroxyl, alkyl, alkoxy or chlorine, attached at specific positions, can confer different biological activities and genotoxicity profiles [33,34,35]. Lucidin (**2**) and rubiadin (**3**), which are 1,3-dihydroxyanthraquinones possessing a methyl or hydroxymethyl group at carbon 2, showed strong mutagenicity, whereas alizarin (**1**), 1,2-dihydroxyanthraquinone without other substituents, had no mutagenicity. Further, 4,5-dihydroxylation of 2-methyl anthraquinones did not have any apparent effect on the mutagenicity. The results suggested that both the number of OH-groups and their positions are relevant for the mutagenicity [32].

Many studies have found antimicrobial properties in biocolourants [36,37,38]. When testing toxicity in vitro, it is important to analyse the conditions and observed changes in detail to distinguish between toxic and non-toxic antimicrobial activity. Antimicrobial activity can be considered toxic (i.e., cytotoxic) if it induces changes at the level of cell structure [39].

### 1.3. Flavonoids and Carotenoids in Tanacetum Vulgare

*T. vulgare* L., commonly called tansy (Table 2), is a flowering herbaceous plant widely distributed in the Northern Hemisphere and known for its curing properties for colds and fevers [39].

Tansy has been reported for its variety of chemotypes based on the most predominant constituent in the essential oil, which may change according to its growth stage [16]. In addition to typical monoterpenes, less volatile sesquiterpenes, sesquiterpene lactones, non-volatile terpenes, flavonoids, and phenolic acids are also present [15,40,41,42,43,44,45]. Flavonoids and carotenoids are the major colouring constituents in *T. vulgare*. For dyeing purposes, the whole plant can be used, resulting in a greenish-yellow colour, while flower heads produce bright yellow [13,46].

In the flower heads, luteolin (**5**), quercetin (**6**), violaxanthin (**7**), β-carotene (**8**) (Table 2) and their derivatives are dominating colourant components [12,13], whereas caffeic, ferulic and neochlorogenic acid and their derivatives occur in all plant parts [12,37,47,48,49]. The amounts of potentially harmful alkaloids and terpenes (e.g., sesquiterpene lactones) depend largely on the chemotype. *Exo*-methylene group in the lactone is regarded to be essential for the cytotoxicity, but also the presence of the O=C-C=CH_2_ system has been shown to be responsible [40,42,50].

Devrnja et al. [37] identified neochlorogenic acid, 3,5-*O*-dicaffeoylquinic acid, dicaffeoylquinic acid and quercetin-3-*O*-glucoside as the main secondary metabolites from the MeOH extracts of tansy flower heads. The extracts exhibited a strong antiproliferative effect on human cervical adenocarcinoma (HeLa) cells causing cell shrinkage and detachment but did not lead to a significant cell detachment of human non-tumour cells (MRC-5) under the same conditions. The tansy was categorised as *trans*-chrysanthenyl acetate chemotype with low proportions (< 10%) of generally harmful monoterpenes *trans*- and *cis*-thujone [37]. The water extract of tansy leaves possessed a strong diuretic action but no renal toxicity or other detrimental effects in tests on rats [51,52,53,54].

### 1.4. Tannins in Salix spp.

*Salix* species (Table 2) are deciduous shrubs, trees or subshrubs occurring mainly in the temperate and arctic areas of the Northern Hemisphere. Leaves and bark are rich in secondary metabolites such as condensed tannins, constituting approx. 2–3% of dry weight (dw) [10,23,55,56] but even 10% (dw) in twigs (*Salix pyrolifolia* Ledeb.) [10]. Tannins bind to proteins and carbohydrates and accumulate alkaloids and metals (e.g., Al, Fe, Cu). Traditionally, willows (*Salix* spp.) have been considered safe, as they have been used as a medicine to reduce fever, inflammation and pain. Adverse effects of bark extracts appear to be minimal. The primary risk may relate to allergic reactions in salicylate-sensitive individuals [57,58]. 

The composition of secondary metabolites varies highly among *Salix* species, between genotypes and hybrids [10,14,56,59,60]. The condensed tannins in willow bark are procyanidins, which consist of chains with varying numbers of flavan-3-ol monomers linked to each other. The bark contains higher amounts of phenolics and higher diversity of glycosides than the leaves [14,59,61,62]. In dyeing, tannins produce browns and can be used as biomordants [13]. 

*Salix* bark contains simple phenolic glucosides like picein (**9**), triandrin (**10**), catechin (**11**) and salireposide (**12**) as main colourants reported by Dou et al. [63]. *Salix* spp. also contain other phenolics, such as phenolic acids, flavones and chalcones and yellow providing flavonols, especially quercetin (**6**) derivatives.

### 1.5. Anthraquinones in Cortinarius Semisanguineus

*Cortinarius* spp. are ectomycorrhizal fungi growing mainly in coniferous forests. Of the secondary metabolites, fungi *Cortinarius*, subgroup *Dermocybe*, are rich in monomeric anthraquinones also containing dimeric pre-anthraquinones of flavomannin type [13,64,65,66,67,68].

The fruiting bodies of *C. semisanguineus* (Table 2) are relatively small (< 10 cm, cap ∅ 3–9 cm), but are produced in large numbers by the mycelia. Among the more abundant compounds are emodin (**13**), emodin-1-glucoside, dermocybin (**14**), dermocybin-1-glucoside and dermorubin (**15**). Also, physcion (**16**), dermolutein, 5-chlorodermolutein, 5-chlorodermorubin, dermoglaucin, endocrocin, and erythroglaucin have been found [68]. Anthraquinones from *Cortinarius* species have inhibited the growth of *Staphylococcus aureus* and *P. auerugosa* [69], and many anthraquinones have bioactive properties [70].

Macromycetes have mostly been studied for their oral toxicity in humans [71]. Highly nephrotoxic bipyridine orellanine, which is a good aluminium ion chelator, has been found in *Cortinarius orellanus* from Europe and *Cortinarius armillatus* native to North America [67,72,73]. Because *Cortinarius* fungi are endemic, it should not be assumed that toxins in European species are similarly present or absent in species of other regions, even if they appear morphologically similar [72,73]. Orellanine has not been found in the European species of *C. armillatus*, nor in *Cortinarius sanguineus* (syn. *Dermocybe sanguinea*) [74] or *C. semisanguineus* [75], which all contain anthraquinones and have been used for dyeing [13].

Toxicity tests for anthraquinones [33,37] show that the structural requirements for their mutagenicity in *Salmonella typhimurium* are not so clear. It has been suggested that the presence of alkyl or alkoxy groups in the anthraquinone structure, e.g., of chrysophanol, emodin (**13**), icelandicin, lucidin-2-ethyl ether, dermoglaucin, catenarin, and cynodontin, results in changes in cell tests [33].

Emodin (**13**) and dermoglaucin were found to be non-toxic for *Bacillus subtilis* in rec-assay and *E. coli* in *pol A* assay. However, dermocybin (**14**) showed very slight mutagenicity for *B. subtilis,* but no toxicity for *E. coli* [33]. There are an increasing number of reports regarding adverse effects of emodin (**13**). It has been reported to induce genotoxicity, reproductive toxicity, and also hepatotoxicity and nephrotoxicity, presumably connected to high doses and long-term use [76,77].

Von Wright et al. [78] studied the genotoxicity of the crude fractions from the extracts of *C. sanguineus* using an in vitro bacterial repair assay (*E. coli*; strains WP2 and CM871), a sister-chromatid exchange (SCE) test and a mouse hepatoma cell assay (Hepa-1c1c7). Emodin (**13**) was the most abundant (44%) compound in Fraction I. Analysis showed that standard emodin was not toxic to either of the *E. coli* strains. However, it induced more SCEs than Fractions I and IV. Fraction I was clearly positive in both SCE- and Hepa-1 tests and in *E. coli* assays, indicating that other emodin-related but yet unidentified compounds were responsible for the observed cytotoxic and clastogenic effects. It was concluded that the different test results had somewhat contrary results and discrepancies existed between SCEs and *E. coli* assay tests results.

### 1.6. Terphenyl Quinones in Tapinella Atrotomentosa

*T. atrotomentosa* (syn. *Paxillus atrotomentosus*) (Table 2) is a sturdy dark brown-capped wood rotting fungus, with a dark brown, velvety stalk, and rim of the unsymmetrical cap. It is common on moss-covered pine stumps in boreal forests, pine moors and forest edges in Europe, North America and Asia [13,38]. The easily harvestable pale flesh fruiting body can grow to a relatively large size (cap ∅ ≥ 20 cm) and is rich in colourants. *T. atrotomentosa* has not proved to be toxic but no dietary use has been recommended [36,79].

The colourants isolated from its fresh fruiting bodies typically have a terphenyl quinone structure [80]. The *p*-terphenyl core (Table 2, **17**) is essentially restricted to fungi and lichens and the structure was found to possess notable antiproliferative, antibacterial, antioxidant, and anti-inflammatory activities [38].

Atromentin (**17**) isolated from *T. atrotomentosa* is responsible for the brown colour. It occurs as colourless precursors, leucomentins 2 and 3 (**18**, **19**) in the flesh [38,80], and its content may be ca. 2% of the fungal mass (dw), those of leucomentins about 5% [80]. Orange-yellow flavomentins and violet spiromentins (**20**) [81,82], osmundalactones and ergostane-type ecdysteroids (e.g., paxillosterone and atrotosterones A–C) have also been found [38,82].

T. atrotomentosa has a broad-spectrum of antimicrobial activity against Gramᐩ and Gram^-^ pathogens, and a significant inhibitory activity against resistant bacterial strains, e.g., MRSA [38]. A recent investigation showed that violet colour providing spiromentins B and C (spiromentin core (**20**), Table 2) have strong antibacterial activity against multiresistant *Acinetobacter baumannii* and *E. coli* [38].

### 1.7. Objectives

Our aim with this paper was to discuss the toxicity of the examined biocolourants, their sources and the dyed textile samples. We evaluated the results of cytotoxicity tests when two plant species, *T. vulgare* and *S. phylicifolia*, and two fungal species, *C. semisanguineus* and *T. atrotomentosa,* were used as natural dye sources. The extracts were obtained directly from plant and fungal material and the wool fabrics dyed with them. Preliminary toxicity screening was performed using Hepa-1 mouse hepatoma cell tests in vitro and examining the highest tolerated dose (HTD) after exposures of 24 and 72 h. The data obtained were analysed by statistical means.

Common natural dye sources *T. vulgare*, *S. phylicifolia, C. semisanguineus,* and *T. atrotomentosa* were chosen due to their high colouring and pharmacological potential, and the information we already had gained of their characteristics and dyeing properties [13,18,46,63,68,83,84]. The characteristic colourants of the studied plants and fungi are described in Table 2.

## 2. Results

### 2.1. Colourants and Colourfastness

**Figure 1:**
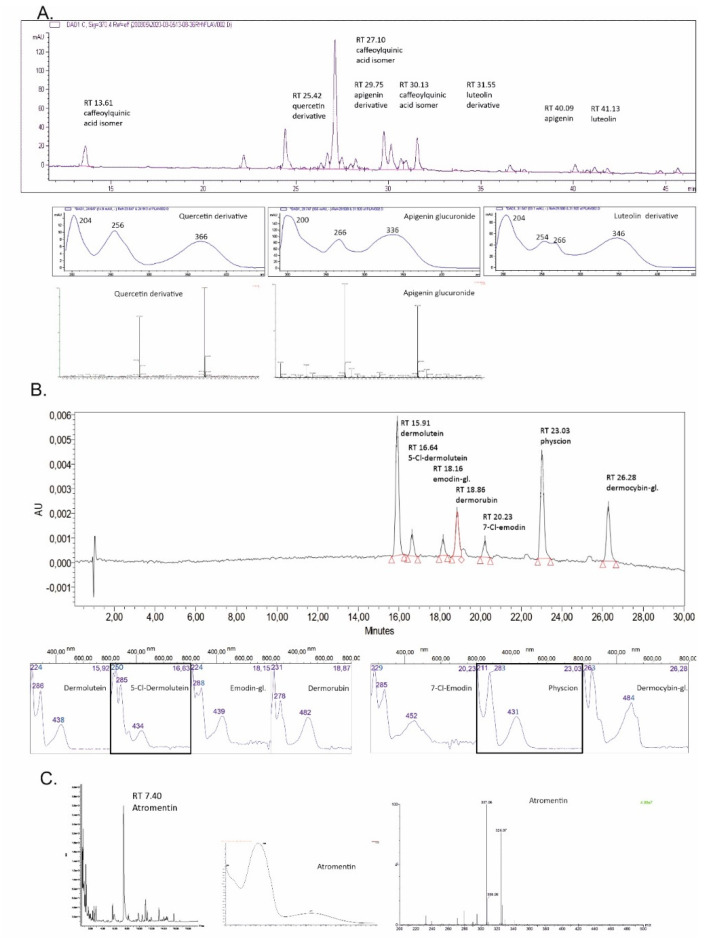
HPLC chromatogram, UV/Vis and MS spectra of the main compounds of (**A**) *Tanacetum vulgare* (HPLC detection at 370 nm), (**B**) *Cortinarius semisanguineus* (HPLC detection at 440 nm) and (**C**) *Tapinella atrotomentosa* (HPLC detection at 370 nm) extracts. Detailed data is in Table 3.

The main colourants of the fungal and plant species under study were tentatively identified and the results can be seen in the HPLC-UV/Vis-MS spectra in Figure 1 and Table 3. Our analysis is in accordance with the previously published works as described in Introduction. The tentative identification of flavonoids (*T. vulgare*) from the MS data revealed the presence of several flavonol and flavone glycosides such as the derivatives of quercetin, apigenin and luteolin. Furthermore, caffeoylquinic acid isomers and dicaffeoylquinic acids were tentatively identified, which is in accordance with the literature [37]. 

In *C. semisanguineus* dermolutein, physcion, dermocybin-1-glucoside, and dermorubin were identified together with three other antraquinones and the results follow our previous findings [68]. In HPLC, *T. atrotomentosa* showed one major peak which was identified as atromentin with the UV/Vis and mass spectrometric data (Table 3).

The colours of the dyed woollen samples are shown in Table 2 and the colour fastness results in Table 4. The results presented in Table 4 show that mordant increases the stability of the dye structure, which can be seen from the higher light fastness values compared to the non-mordanted samples. The use of iron mordant gives slightly higher values, which can be explained by its ability to form stabilising coordination complexes [86]. Washing fastness tests reveal that staining is quite low, but highly basic (pH ~10) washing liquor results in colour changes that are particularly visible in yellow and red hues. In all cases, colour after washing was stronger than before, and its hue was different. This is due to bathochromic change of the dye molecule’s absorption maxima as a result from the ionisation of the free OH-groups. Metal complexes formed by Al^3+^ and Fe^3+^ ions stabilise the structure and decrease colour change as seen from their higher values.

### 2.2. Toxicity

In the HTD tests, the limit of significant toxicity was placed at EC_50_ [52]. Thus, if over 50% of the exposed cells exhibited changes in cell morphology and viability (i.e., HTD value was 3 or 4), the influence of the sample was interpreted as being toxically significant. HTD value 2 was regarded as the result of a low toxic effect, and subsequently, the value of 1 as a non-toxic, harmless effect.

EtOH controls (0.5, 1, 2, 4%) in parallel were first tested on cells. It was found that no cell changes were observed (i.e., HTD values 0) at the EtOH concentrations up to 2% after exposure of 24 h. However, when the EtOH concentration rose to its highest (4%), one of the parallel samples had undergone mild cell changes (< 25%), indicating non-toxic, harmless effect.

After longer exposure time (72 h) with similar parallel EtOH concentrations up to 2%, no cell changes were observed. However, with the highest EtOH concentration (4%), the quantity of cell changes rose to the next level (< 25%), similarly than in the shorter time of exposure, but now both of the parallel samples had undergone changes, indicating non-toxic effect.

Although the quantity of cell changes (< 25%) with the HTD value 1 had been regarded as harmless by Klemola (2008), excluding any possible toxicity caused by the solvent was wanted, and therefore, EtOH concentrations 0.5, 1 and 2% were selected for further inspections.

#### 2.2.1. The Effect of EtOH Concentration on the Cell Morphology and Viability

**Figure 2:**
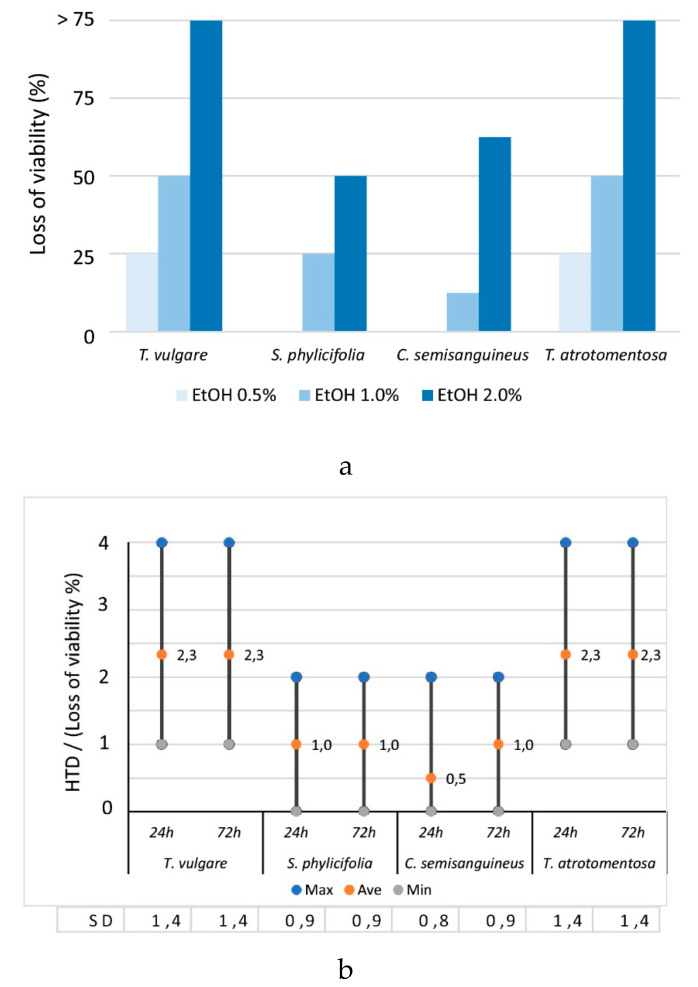
The Toxicity of the Plant and Fungal Materials. (**a**) The effect of EtOH concentration on the average changes in the morphology and the loss of viability% for the different EtOH dilutions. Samples of 24 h and 72 h exposure times are included. (r_s_ = 0.863, *p* < 0.0005, n = 192). (**b**) The effect of time on the HTD values (changes in morphology and loss of viability (%) where 1 < 25%, 2 = –50%, 3 = –75%, 4 > 75%). The ranges and average values are marked in the columns and standard deviations (SD) at the bottom. The results (n = 195) include EtOH concentrations of 0.5, 1 and 2%.

EtOH extracts were diluted to the concentrations of 0.5, 1 and 2% EtOH with α-MEM. The results showed the stronger the EtOH concentration was, the higher the amount of change in the morphology and the loss of viability% (HTD value) in exposed cells, showing a highly significant correlation (Figure 2a).

#### 2.2.2. The Toxicity of the Plant and Fungal Materials

None of the dye sources (*Tanacetum*, *Salix*, *Cortinarius* and *Tapinella)* were significantly toxic when the EtOH concentration was 1% or less. This indicated that 1% EtOH contained extracted compounds in the amounts and composition, which was some kind of threshold value. The changes in the cell morphology and the losses of viability, which the diluted 1% EtOH extract induced, were < 50% (i.e., HTD value was 2 at the maximum).

According to our results, *Tanacetum* and *Tapinella* extracts were the most toxic samples. If the extracts were diluted to 2% EtOH, they caused changes in the cell morphology in the quantity of 75% (i.e., HTD values were 4), indicating significant toxicity (Figure 2a).

Time did not have any effect on toxicity i.e., the cell morphological changes were at the same quantities (Figure 2b) when comparing exposure times of 24 h and 72 h of plant and fungal material. However, there were slightly more cell changes with increased exposure time caused by *Cortinarius* extracts, but the difference was statistically insignificant compared with the other samples (Mann–Whitney U-test, *p* > 0.05, n = 44).

#### 2.2.3. The Toxicity of the Undyed Fabrics

Unmordanted and mordanted undyed fabric samples diluted to 0.5% EtOH did not show any changes indicating non-toxicity (i.e., HTD value was 0). However, when these samples were diluted to 1% and 2% EtOH, exposed cells showed changes in morphology and viability in the quantity of < 50%, indicating a low toxic effect at the maximum.

**Figure 3:**
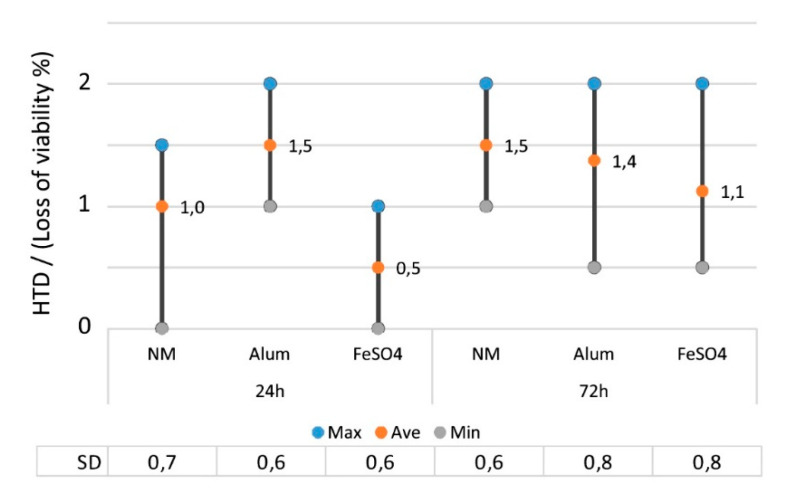
The effect of time on the changes in the morphology and the loss of viability (%) for the mordanted but undyed fabrics, where the HTD value of 1 indicated < 25% loss of viability and 2 between 25–50%. The results contain the values of 1 and 2% EtOH dilutions. NM = no mordant, SD = standard deviation.

A longer time of exposure slightly increased changes in exposed cells, but the results were still below toxicity, i.e., average HTD values were 1.0 after exposure of 24 h, and 1.3 after 72 h (Figure 3). The difference between the two timespans was statistically insignificant (Mann–Whitney U-test, *p* > 0.05, n = 24).

Iron (FeSO_4_) mordanted fabric samples induced the least cell changes, as HTD values were 2 at the maximum indicating from non-toxic to low toxic effects, but the differences compared to alum (KAl(SO_4_)_2_) or unmordanted samples were insignificant (Kruskal–Wallis-test, *X*^2^ = 3.37, *p* > 0.05, n = 24) (Figure 3).

#### 2.2.4. The Toxicity of the Dyed Fabrics

**Figure 4:**
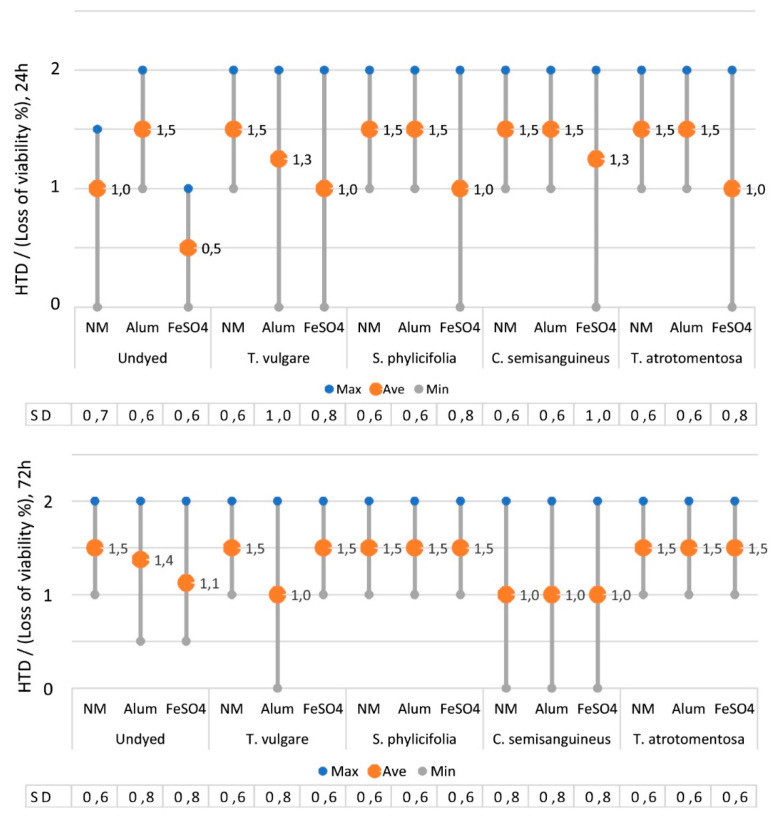
The effect of dye source, mordant and time on the HTD values (changes in morphology and loss of viability%) after 24 h (above) and 72 h (below) exposure times. EtOH dilutions of 1% and 2% are included (n = 152). NM = no mordant, SD = standard deviation.

The CIELab values of the colours obtained from dyeing are shown in Table 2. The dyed fabric samples induced slightly more changes to exposed cells than undyed fabric samples, but the differences were insignificant (Kruskal–Wallis test, *X^2^*(1.120) = 1.339, *p* > 0.05 (*p* = 0.247), n = 360) (Figure 4). 

When the dyed fabrics were diluted to 0.5% or 1% EtOH, their effect on the morphology and viability of the exposed cells was < 25%, i.e., HTD values were 1 at the highest, indicating the diluted dyed fabric samples were non-toxic.

All the dyed fabric samples caused changes on exposed cells when they were diluted to 2% EtOH. In that case, at least one of the two parallel samples induced 25–50% losses of morphology and cell viability, i.e., HTD values were 2, indicating a non-significant but still low toxicity.

Iron mordanted dyed samples induced the least changes on exposed cells i.e., low toxic effects at the maximum, after exposure of 24 h. However, after exposure of 72 h, the differences between iron mordanted dyed samples and undyed controls became smaller, and the iron mordanted samples obtained HTD values similar to all others (Figure 4).

In the case when all the samples were examined, it was observed that the dyed fabrics induced slightly more changes to exposed cells than the undyed fabrics. Those changes indicated harmless, non-toxic, or low toxic effects at the maximum. However, the difference between these two groups was statistically insignificant (Kruskal–Wallis test, *X^2^* = 1.34, *p* > 0.05, n = 192).

The length of the exposure time did not increase the difference in inducing cell changes caused by the dyed fabrics compared to the undyed controls.

Interestingly, for diluted undyed fabric samples and diluted *Tanacetum-*, *Salix-* and *Tapinella*-dyed fabric samples, effects on exposed cells increased with time, whereas for *Cortinarius* the situation was contrary: The longer the exposure time, the fewer changes were observed in the exposed cells. This was shown by the lower HTD values after the exposure of 72 h.

#### 2.2.5. The Effect of Mordant on the Toxicity

The effect of the mordant on the colour can be seen of the CIELab values in Table 2. Iron mordant obviously darkened colours.

The analysis showed that iron mordant induced the fewest changes to exposed cells compared to other mordants, as shown by the lowest HTD values (Figure 4). In all the samples there was slightly more variation (max and min values) observed after the exposure time of 24 h than 72 h, showing time evening out the differences.

Unmordanted but biocoloured fabrics (*Tanacetum*, *Salix* and *Tapinella*) did not produce any difference in the HTD values at either exposure times; nor did they produce any difference compared to the unmordanted undyed fabric sample. When examining the HTD values of unmordanted samples after the exposure of 24 h, it was observed that the dyed fabrics received slightly higher values than the undyed controls, but after 72 h, the differences between dyed and undyed fabrics had disappeared. The only exception was *Cortinarius,* which received lower HTD values after 72 h than 24 h, with all mordants.

The alum mordanted *Tanacetum* fabric samples differed slightly from other dyed samples and the undyed controls by causing fewer cell changes at both exposure times, the difference being statistically insignificant.

The samples that were mordanted with iron and dyed with different biocolourants did not differ significantly from each other.

When examining differences between the mordants, it was observed that iron induced the least change in exposed cells, although the differences compared to other mordants were insignificant (Kruskal-Wallis, *X*^2^ = 3.33, *p* > 0.05, n = 120). The most changes were observed after the exposure of 24 h, but the Kruskal–Wallis test revealed that the differences between all the mordants were insignificant (*X*^2^ = 5.73, *p* > 0.05, n = 60).

## 3. Discussion 

The sample extracts (99.5% EtOH) were diluted to three EtOH concentrations (0.5, 1.0, 2.0%) and the results showed that the higher the EtOH concentration, the more changes in the morphology and viability of exposed cells were observed. This was likely because of the increase in the concentrations of compounds extracted with EtOH from the plant and fungal material.

With the lowest EtOH concentration, no cytotoxicity was observed, indicating that the type and number of toxicity-inducing compounds in the sample was low. However, further studies would be needed to analyse these compounds, their character and behaviour more precisely.

Both the plant and fungal extracts and dyed fabrics of *Tanacetum* and *Tapinella* showed the most change in the morphology and viability of the exposed cells. If their extracts were diluted to 2% EtOH, the changes were toxically significant. 

The studied sample of *T. vulgare* (Finland) was a camphor chemotype (71% camphor), but had no quantifiable thujones [18,41], which are regarded at high concentrations to exhibit toxicity [40]; so, we suppose there are some other components than thujones that are responsible for the observed low to moderate cytotoxicity. Furthermore, the tansy used in this research was collected and dried years ago and long storage time may have affected the composition and content of colourants and other compounds. Our qualitative HPLC-UV/Vis-MS analysis showed that the majority of the secondary compounds had preserved well over the years.

According to previous studies that used the same solvent as we did, i.e., EtOH, or similar EtOH:H_2_O, extracts obtained from the aerial parts of *Tanacetum* species contained secondary metabolites from the groups of sesquiterpene, non-volatile sesquiterpene lactones, coumarins, phenolic acids, and flavonoids [49,87,88,89]. Previously, the essential oils of tansy have shown cytotoxicity. The oil distilled from the aerial parts of *T. vulgare* (Canada) with camphor (31%), borneol (15%) and 1,8-cineole (i.e., eucalyptol) (11%) as the major components were slightly cytotoxic against human healthy cell line WS1 and human keratinocytes (HaCaT). Furthermore, its minor components, α-humulene (0.21%) and caryophyllene oxide (1.13%), possessed a moderate cytotoxicity against both of these [90].

The recent results [44] strongly implied that the toxicity of *T. vulgare* (β-thujone chemotype) essential oil, obtained from its aerial parts with hydrodistillation, should not be associated exclusively with thujones. The tests using the *Artemia salina* model indicated that some other (minor) constituents of the oil were responsible for the observed more pronounced acute toxicity. For example, sabinene, terpinen-4-ol and caryophyllene oxide showed very strong negative correlations (r > −0.8) with the live nauplii after 48 h, while all other constituents (e.g., linalool, intermedeol, sabinyl acetate) showed strong (r > −0.6) negative correlations [44]. Previously, it has been shown that a linalool rich oil was more toxic in this brine-shrimp bioassay than the sample that did not contain this monoterpene alcohol [44].

Rosselli and coworkers [42] isolated five sesquiterpene lactones with the eudesmanolides skeleton from the dried flowers of *T. vulgare* ssp. *siculum* (Guss). All of them (douglanin, ludovicin B, ludovicin A, 1α-hydroxy-1-deoxoarglanine, 11,13-dehydrosantonin) had high time- and concentration-dependent cytotoxic activity against in vitro cultured cancer and healthy cell lines (V79379A), being more effective against the latter ones.

As far as we are aware, there have been no previous cytotoxicity studies concerning *T. atrotomentosa,* which would have shown significant toxicity when the extract was diluted to 2% EtOH. Despite this, its chemistry holds great potential, as atromentin, the central terphenylquinone intermediate of a large family of homobasidiomycete secondary metabolites, represents the generic precursor molecule for the entire terphenylquinone and pulvinic acid family of colourants.

Atromentin may be modified by oxidative ring splitting into atromentic acid (yellow), or by dihydroxylation and symmetric heterocyclisation to thelephoric acid; or by reduction and esterification to produce leucomentins, which can upon saponification, release osmundalactones [91].

The terphenylquinone compounds have gained pharmaceutical attention due to their multiple bioactivities and have been produced under laboratory conditions [91]. The significant cytotoxicity we found in these preliminary studies would need further research. *T. atrotomentosa* holds great potential as a source of colourants, but also due to its manifold other properties. In recent years, a lot of effort has been focused on the development of cultivation of fungi for colourant production [11,92,93].

For *Salix* and *Cortinarius,* the HTD values caused by their extracts and dyed fabrics were quite similar. Both showed low cytotoxicity when diluted to 2% EtOH. The observed toxicity changes did not alter after exposure of 72 h.

The compounds and interactions that lie behind the observed toxicity of *Cortinarius* extracts are probably due to anthraquinones, the chemistry and toxicity of which were found in previous studies, are presented in Section 1.5. Natural anthraquinones are interesting compounds in many applications and they need more detailed studies.

The low cytotoxicity of *Salix* extracts was presented for the first time. Generally, the extracts of *Salix* bark have been regarded as being safe, although sensitive individuals may get allergic reactions due to salicylates. Studies have also shown that there are other salicin-related compounds that contribute to various effects, which may also concern cytotoxicity [57].

Textile production procedures are complex. During the procedures, many different auxiliary chemicals are used, and extracts may contain remnants of them. In our study, the undyed fabrics were not interpreted as being cytotoxic; however, they caused effects from non-toxic to low toxic, depending on the concentration of EtOH, for both unmordanted and mordanted fabrics, indicating that it is also important to analyse the toxicity of the undyed fabric itself.

All unmordanted and alum (KAl(SO_4_)_2_) and iron (FeSO_4_) mordanted fabrics showed slight changes (≤ 50%) in cell structures when diluted to 1 or 2% EtOH and interpreted toxically insignificant changes. The study showed that iron mordanted fabric samples induced the least cell changes, but the differences compared to alum (KAl(SO_4_)_2_) or unmordanted samples were insignificant. The reason for this is not known, but leucomentins of *Tapinella* may act as iron chelators [94], and condensed tannins of *Salix* species form complexes with metal ions [60].

In these cell tests, the concentration of plant/fungal material was approx. 50–800 μg/mL of α-MEM. If organic material contains approx. 4% of biocolourants, a maximum of 2–32 μg pure dye/mL of α-MEM was used in the cell tests. This is considerably less compared with Klemola [52] who used 50–1600 μg pure synthetic dye/mL medium in the studies. In the future studies, higher concentrations of plant/fungal material should be tested.

Using pure dyes in tests would give less ambiguous results. On the other hand, it is difficult to predict the toxicity of a dyed material from just testing the dye, because one chemical itself may be non-toxic but the chemicals and materials together may have a totally different effect [95]. Also, differences exist between a plant material extract which contains all soluble compounds and an extract from a dyeing when only the portion of substances which had been adsorbed during dyeing and released in later extraction are present. Development of biocolourants would be more economic if crude extracts were used instead of fractions or purified compounds. If additional values of biocolourant are to be developed, it is essential to consider whether the product would be purified or a mixture, as in the purification step, activities may be lost or decreased [96].

The cell tests can give information about the potential toxicity of extracts from textile materials. However, the cell type and test method used have an impact on how the analysis results may be interpreted. Only one type of cell, Hepa-1, was used in this research, and conclusions are only directly applicable to this cell type. The cases in which one cell line analysis shows slight cytotoxicity may appear to be harmless to humans. To draw a more reliable picture, several different cell lines should be used, and the total impact evaluated based on all results. Nonetheless, Hepa-1 cells have been observed to predict potential cytotoxicity in humans better than rat cells [52]. Hepa-1 cytotoxicity test can give useful information about the overall toxicity of extracts from textiles and can help in the development of safe textile products [97].

When considering the harmful effects of dyes, discussion is most often focused on environmental effects and the elimination of dyes from textile effluents, instead of the direct influence of textile dyes on human health [5]. The biocolourants are biodegradable, but their production, usage at a larger scale, and safety needs much more research.

We tested four extracts of plant/fungal sources and dyed fabrics to answer the question whether it is safe to wear natural dyed textile. The amounts of natural colour compounds in textile after dyeing are low. Washing fastness tests reveal that minimal amounts of colourants were removed during these simulated standardised use phases. This preliminary study showed that risk for harmful effects of natural dyes when wearing textiles are low among the tested dye sources.

## 4. Materials and Methods 

### 4.1. Plant and Fungal Material

Tansy flower heads (*Tanacetum vulgare* L.), obtained from Natural Resources Institute Finland (Jokioinen, Finland), were harvested in 2000. The plant previously studied by Keskitalo [18] was classified as camphor chemotype. Flowers were dried and stored in the dark at room temperature (RT) until used in June 2010. A 1:1 mass ratio of dried flower heads to textile material was used for the dye liquor.

Willow (*Salix phylicifolia* L.) was gathered from Sipoo, Finland, in May 2008. Bark was separated and left to dry in a dark place at RT until used in June 2010. To prepare the dye liquor, a 5:1 mass ratio of dried bark to textile material was used. The bark was soaked in water for 2 days before heated extraction.

Fruiting bodies of surprise webcap [*Cortinarius semisanguineus* (Fr.) Gillet] were collected from Jyväskylä, Finland, in 2005. They were dried at 60 °C and stored in a dark place at RT until used in June 2010. To prepare the dye liquor, a 1:1 mass ratio of dried fungi to textile material was used.

Velvet roll-rims [*Tapinella atrotomentosa* (Batsch.) Šutara] were gathered from Espoo, Finland, in September 2009 and deep-frozen until used in June 2010. To prepare the dye liquor, a 10:1 mass ratio of defrosted fungi to textile material was used.

The preparation of dye liquors followed the traditional dyeing recipes in which 1:1 dried plant/fungi for textile material and 10:1 fresh fungi/textile material were used. For *Salix*, a five times greater amount of material was used to obtain an identically deep colouration of textile [13].

### 4.2. Methods for Compound Identification

Qualitative analysis of the main colourants of each studied species was performed using HPLC combined with UV/Vis and MS detections. 

The phenolic compunds in *Tanacetum* flowers were analysed by HPLC-DAD and UHPLC-QTOF MS/MS analytical equipments and methods described in Pihlava et al. [98]. Briefly, 300 mg of ground sample was extracted with 10 mL of MeOH for 1 h in an ultrasonic water bath. An aliquot of extract was filtered through a 0.20 mm membrane filter into an autosampler vial for the analysis.

Anthraquinones of *Cortinarius* were analysed with methods described earlier by Vanden Berghe et al. [99].

The ground fungal material of *Tapinella* (100 mg) was extracted with 1 mL of MeOH using ultrasonication for 15 min where after centrifuged and the liquid phase was transferred to another tube. The material was re-extracted twice with 1 mL of MeOH; extracts were combined and evaporated to dryness. The samples were reconstituted in 0.2 mL of 50% aqueous MeOH. Analysis was performed on an Acquity UHPLC system (Waters, Milford, MA, USA) equipped with a Waters Synapt G2-S MS system and Waters Photodiode Array (PDA) Detector. The analytical column ACQUITY UPLC BEH HSS T3 (1.8 µm 2.1 × 100 mm) was kept at 30 °C. The experiment was carried out at a flow rate of 0.4 mL/min with mobile phase A (0.1% formic acid in water) and B (0.1% formic acid in MeOH). The gradient elution started at 20% B (0–0.2 min), was increased linearly to 100% B within 28 min and after this returned within 0.1 min to initial percentage where it was maintained for 2 min. The injected sample amount was 2 μL. Mass spectrometry was carried out using electrospray ionization (ESI) in positive polarity, and leucine enkephalin was used as the lock spray reference compound. The capillary voltage was 2.0 kV, cone voltage 40 kV, source temperature 150 °C, and desolvation temperature 500 °C. The cone and desolvation gas flow were set at 60 L/h (nitrogen) and 1000 L/h (nitrogen), respectively. The data was collected at a mass range of *m/z* 50–800 with a scan duration of 0.2 sec. The PDA detection wavelength range was 200–500 nm.

### 4.3. Dyeing of Fabric Samples and the Colour Fastness Testing

Technical grade alum (KAl(SO_4_)_2_) and FeSO_4_ (TetriDesign, Helsinki, Finland) and tap water (pH ~7.8; Helsinki Region Environmental Services Authority HSY, Helsinki, Finland) were used for dyeing. Pre-mordanting and dyeing were performed in an Original Hanau Linitest machine (Hanau, Germany). The colour was measured as CIE L*, a* and b* values [100] using a Konica Minolta (Tokyo, Japan) CM-2600d spectrophotometer (D65, 10°). Unbleached 100% wool fabric (plain weave, warp and weft densities 6.5 yarns/cm, 84 g/m^2^) (HAMK/Wetterhof, Hämeenlinna, Finland) was made of the SportLoden yarn (Nm 28/2, with Öko-Tex 100 and Bluesign labels) (Schoeller, Germany).

To prepare the dye extracts, the amount of dried or defrosted plant or fungal material, as in Section 4.1, was weighed, chopped or crushed, and boiled in water covering the organic material. After boiling (80−90 °C, 60 min), the extract was filtered. Organic material was discarded and the filtrate used as a dye liquor.

The fabric samples (á 20 g) were pre-mordanted with 10% on the weight of the fibre alum or 2% FeSO_4_ in a 1:40 material to liquor ratio. The mordant was diluted in water, wetted fabric was added and the vessel closed. The temperature was increased to 80 °C and held there for 60 min, continuously agitating the liquid. The samples were squeezed, left to dry at RT and stored in the dark until used. The pH for alum mordant liquor was 3.3–3.9 and for FeSO_4_ 4.5–5.3. The samples without mordant were treated similarly as the pre-mordanted samples. 

The volume of the prepared dye extract was measured, and water was added to meet the 1:20 ratio for dyeing. Pre-mordanted samples were dyed at 90 °C for 60 min and then rinsed until no colour was observed in the water. The samples were left to dry at RT. The colour of each sample was measured as CIELab coordinates (L*, a*, b*) (Table 2). L* obtains values from zero for black to 100 for white; a* > 0 describes the redness, a* < 0 the greenness, b* > 0 the yellowness, and b* < 0 the blueness of the colour [100]. Colour fastness to light and laundering were studied according to the ISO 105-B02/A1-1994 and ISO 105-C06-1997 standards, respectively (Table 4).

### 4.4. Preparation of Plant, Fungal and Fabric Extracts for Toxicity Testing

Plant or fungal material was ground in a mortar (*Tanacetum* and *Cortinarius*) or cut into small pieces (*Salix* and *Tapinella*) and 1.5 g was placed in a 100 mL flask; 37.5 mL EtOH (99.5%, Altia, Helsinki, Finland) was added and the materials were extracted for 24 h at RT. Thereafter, the extract was filtered and refrigerated until use. The colour of the *Tanacetum* extract was light green, *Salix* bright green, *Cortinarius* orange, and *Tapinella* black.

The dyed woollen fabrics and undyed controls were cut into 1 cm × 1 cm pieces and treated similarly to the plant/fungal samples.

### 4.5. Cytotoxicity Assays 

The cell tests were performed as described earlier [53,101]. The effects of each sample extract on the morphology and viability of the cells were evaluated using phase contrast microscopy by observing possible alterations in cell shape, e.g., cell rounding, swelling, cell shrinkage, vacuolization, growth inhibition, attachment, cell lysis, and cell death. Hepa-1 cells containing cytochrome P4501A (Department of Physiology, University of Kuopio, Kuopio, Finland) from mouse liver were used in HTD tests.

Cell culture reagents were α-MEM (Minimum Essential Medium) without glutamine, endotoxin-free FBS (Fetal Bovine Serum), penicillin-streptomycin solution 100×, l-glutamine 100× (200 mM), PBS (Phosphate-Buffered Saline, pH 7.2) 10× without Ca^2+^ and Mg^2+^, and trypsin 0.05%/EDTA (ethylenediaminetetraacetic acid) 0.02% in PBS (EuroClone, Milan, Italy).

EtOH was used as a solvent control in concentrations of 0.5, 1 and 2% and α-MEM as a negative control and solvent medium. Hepa-1 cells were grown in α-MEM supplemented with 1% l-glutamine, 10% endotoxin-free FBS and 1% penicillin-streptomycin solution.

A short-term cytotoxicity test was carried out in 96-well microplates. Hepa-1 cells were left to grow for 2–3 days in cell culture flasks. Erythrosin B (Sigma, Darmstadt, Germany) was used to release the cells from the flasks: a 1:4 dilution was prepared using a 20 μL volume of cells and that of 80 μL of Erythrosin B, which were adjusted to the concentration of 5 × 10^4^ cells/mL. A volume of 200 μL from this dilution was pipetted into each well, resulting in 10,000 cells/well. A Bürker counting chamber measured the number of cells and cell density in each well.

Cells were exposed to the extracts prepared as explained in Section 4.4. EtOH extracts were diluted to the concentrations of 0.5, 1 and 2% with α-MEM and a sample (200 μL) was pipetted in a well. Non-exposed cells with α-MEM were used as negative controls. DNP (2,4(α)-dinitrophenol, pH 2.8−4.7, Merck, Kenilworth, NJ, USA) was used as a positive control and was diluted in DMSO (dimethylsulphoxide, pH 2.8−4.7, Riedel-de Haën, Seelze, Germany). A stock solution of DNP (100 mg/mL) was diluted with α-MEM to the concentrations of 0.5, 0.05 and 0.005 mg/mL. All results were compared with the controls.

After exposures of 24 and 72 h, the cells were washed twice with PBS-buffer. The possible alterations of cells were observed with phase-contrast microscopy and compared to the control cells, which were without exposure. The alterations were described and the quantity of changes was evaluated with a five- step-scale: (0) no changes, (1) < 25%, (2) 25−50%, (3) 50−75%, and (4) > 75% of cells had changed.

Each plant, fungal and fabric sample was studied using 0.5, 1 and 2% concentrations of EtOH extracts diluted with α-MEM and incubation times of 24 h and 72 h as two parallel samples in different places in the 96-well microplates. Four parallel wells were studied from each sample. The results of parallel wells were reported as a single value. These values (1−4) were the basis for the statistical analysis. For a few samples, two values were reported (e.g., 0/1); in these cases, the mean value (0.5) was used for statistical calculations.

### 4.6. Statistical Analyses

The SPSS program (IBM SPSS Statistics for Windows, Version 24.0, Armonk, NY, USA) was used for quantitative data analysis. Statistical significance was placed at *p* < 0.05. The differences were evaluated using non-parametric Kruskal–Wallis one-way analysis of variance and the Mann–Whitney U-test.

## 5. Conclusions

The dilution concentration was shown to be of high importance concerning the toxicity issue. The highest HTD values were observed when the samples were diluted to 2% EtOH concentration, which is due to higher concentrations of compounds including biocolourants. EtOH was used because it dissolves polar and somewhat lipophilic biocolourants, as well as those of potential toxins. Pure 2% EtOH was observed as being harmless.

The most cytotoxic effects were induced by the extracts of *Tanacetum* and *Tapinella*. With 2% EtOH, the HTD values were at the highest 4, which means that over 75% of the tested cells had changes in morphology and loss of viability, indicating significant toxicity; the extracts of *Salix* and *Cortinarius* induced barely perceptible cytotoxicity when diluted to the same EtOH concentration. 

When examining all the data, *Salix* induced the least toxic effects of the tested biocolourant sources. *Tanacetum* showed some toxicity, but the effect declined when the time of exposure increased from 24 h to 72 h. Interpretation of the results could be more feasible if there were standardised methods for biocolourant production, extraction and in vitro testing.

In this research, *Salix* and *Cortinarius semisanguineus* were at most mildly harmful. *Tapinella atrotomentosa* and *Tanacetum vulgare* induced the most changes. At the highest non-toxic EtOH concentrations (2%), all dyed fabrics were interpreted as being not significantly toxic. It seems that biocolourants may be a less harmful alternative to dyes. In all, the cytotoxicity of samples dyed with natural dyes did not differ from untreated wool. When developing the larger scale utilisation of biocolourants, profound bioassays and chemical analyses are necessary from single purified compounds to track the possible hazardous pathways of dyes in the human metabolism and environment. 

## Figures and Tables

**Table 1:** Chemical structures of some natural anthraquinones.

Chemical Compound	Structure
Alizarin (**1**) C_14_H_8_O_4_ M = 240.21 g mol^−1^	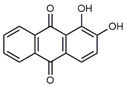
Lucidin (**2**) C_15_H_10_O_5_ M = 270.24 g mol^−1^	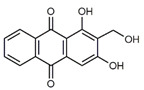
Rubiadin (**3**) C_15_H_10_O_4_ M = 254.24 g mol^−1^	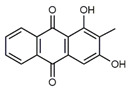
Xanthopurpurin/ Purpuroxanthine (**4**) C_14_H_8_O_4_ M = 240.21 g mol^−1^	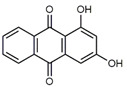

**Table 2:** Sources used in this research, their main colouring compounds as aglycones and dyeing results on wool as CIELab coordinates L, a*, b*. (Photos: Riikka Räisänen, except *Salix phylicifolia* by Jouko Rikkinen [74]).

Scientific Name	*Tanacetum vulgare* L.	*Salix phylicifolia* L.	*Cortinarius semisanguineus* (Fr.) Gillet	*Tapinella atrotomentosa* (Batsch.) Šutara
Common names	Tancy, golden buttons	Willow, tea-leaved willow	Surprise webcap	Velvet roll-rim
Appearance	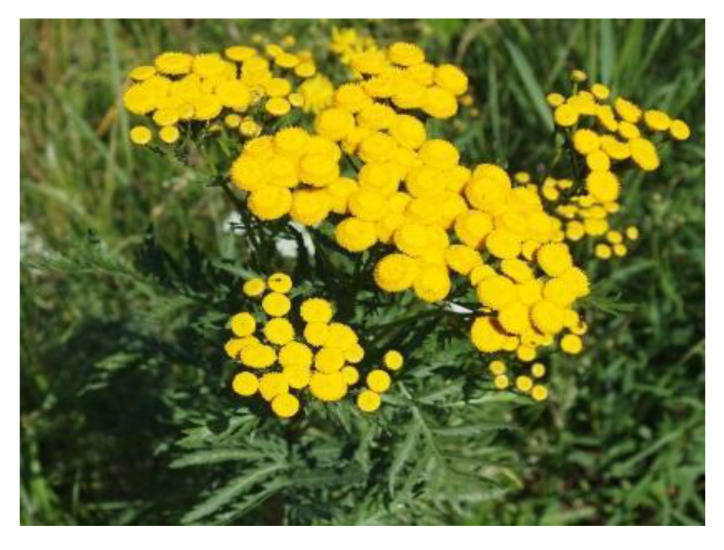	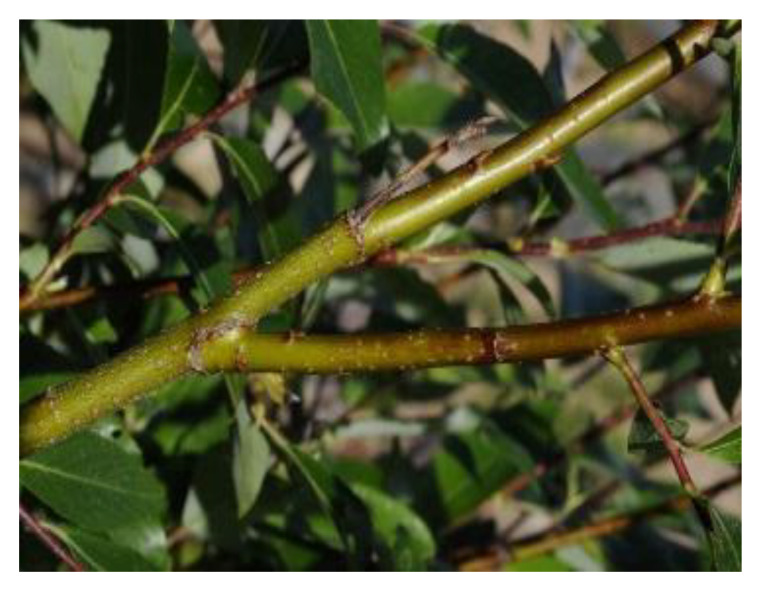	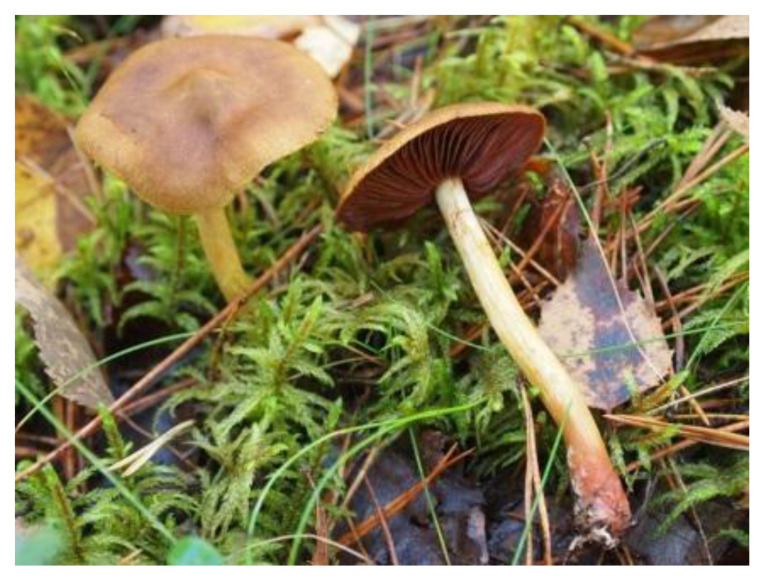	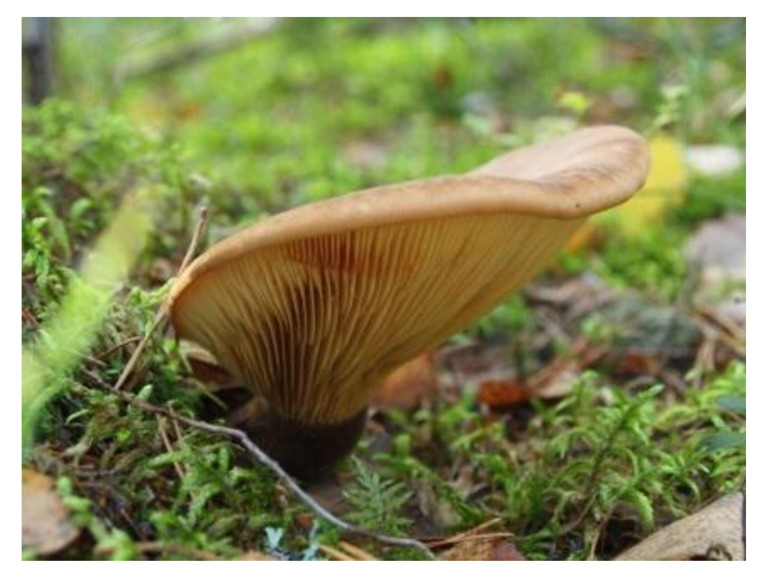
CIELab coordinates	L 70.74; a* 2.54; b* 29.60	L 56.39; a* 13.43; b* 24.98	L 49.90; a* 21.22; b* 20.14	L 38.64; a* 4.07; b* 18.27
Direct dye, NM				
	L 68.66; a* 3.30; b* 49.18	L 57.09; a* 12.58; b* 26.54	L 43.65; a* 34.49; b* 30.05	L 33.68; a* 3.42; b* 16.50
KAl(SO_4_)_2_				
	L 35.07; a* 0.45; b* 13.64	L 43.81; a* 5.06; b* 15.58	L 35.62; a* 8.84; b* 10.63	L 23.63; a* -0.24; b* 10.66
FeSO_4_				
Main colourants [ref]	[12,13]	[63]	[64,66,68]	[79,80]
	Luteolin (**5**) 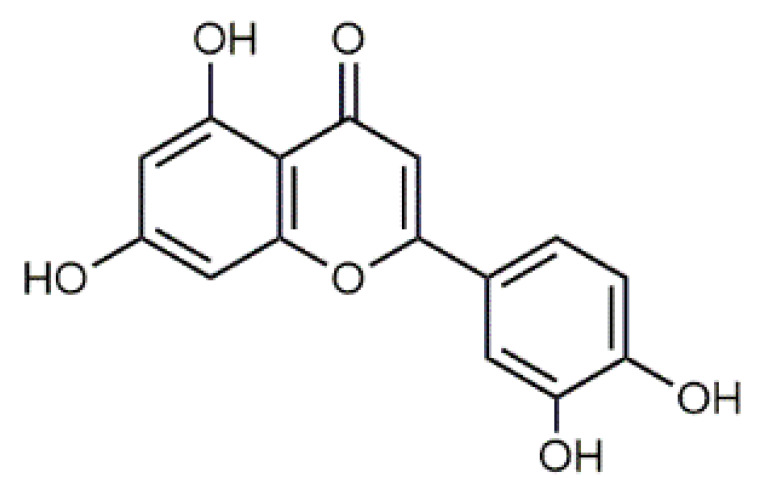 C_15_H_10_O_6_ M = 286.24 g·mol^−1^	Picein (**9**) 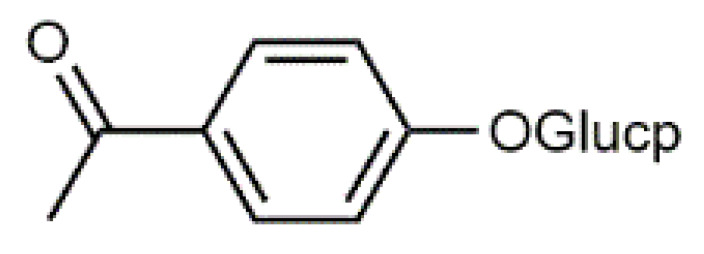 C_14_H_18_O_7_ M = 298.29 g·mol^−1^	Emodin (**13**) 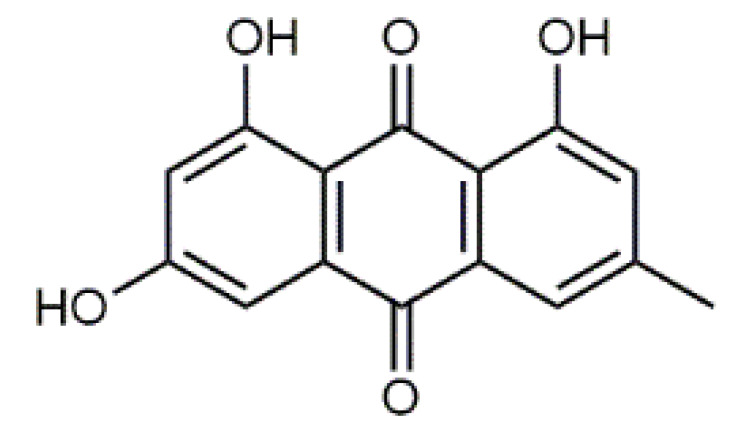 C_15_H_10_O_5_ M = 270.24 g·mol^−1^	Atromentin (**17**) 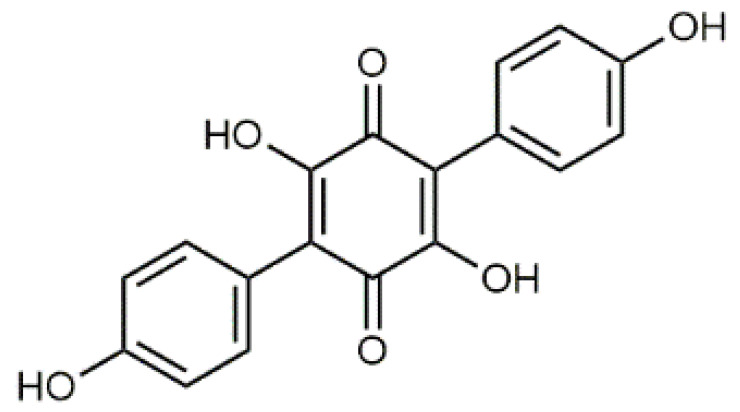 C_18_H_12_O_6_ M = 324.29 g·mol^−1^
	Quercetin (**6**) 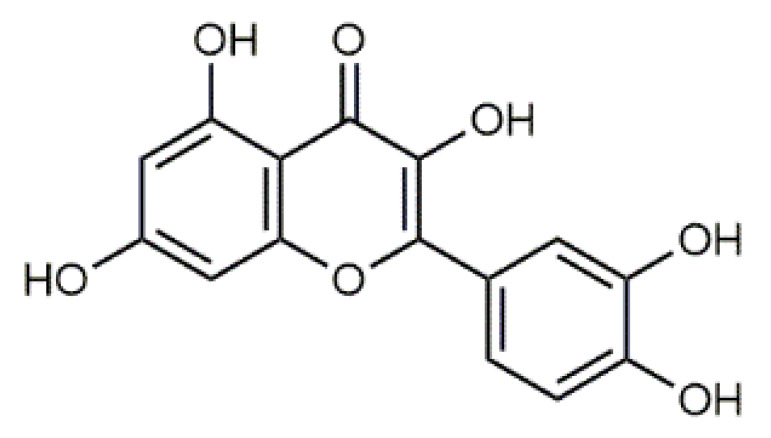 C_15_H_10_O_7_ M = 302.24 g·mol^−1^	Triandrin (**10**) 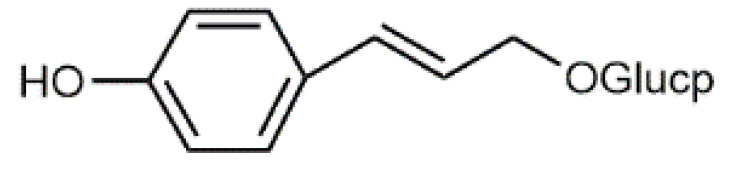 C_15_H_20_O_7_ M = 312.31·g mol^−1^	Dermocybin (**14**) 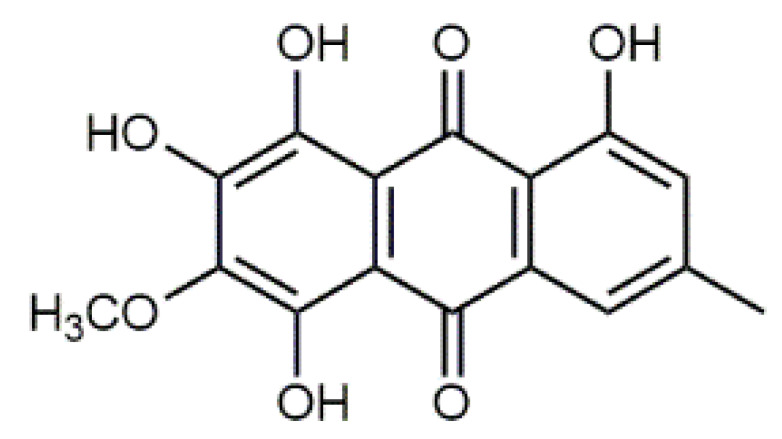 C_16_H_12_O_7_ M = 316.27 g·mol^−1^	Leucomentin 2 (**18**) 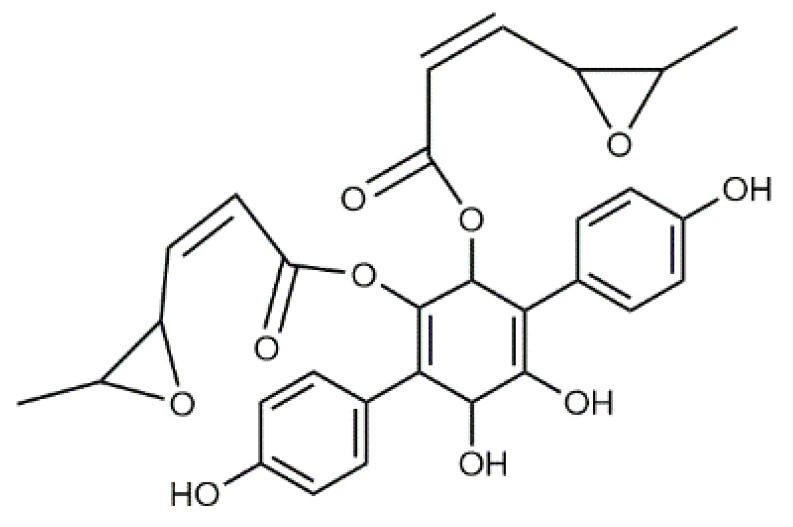 C_30_H_28_O_10_ M = 548.54 g·mol^−1^
	Violaxanthin (**7**) 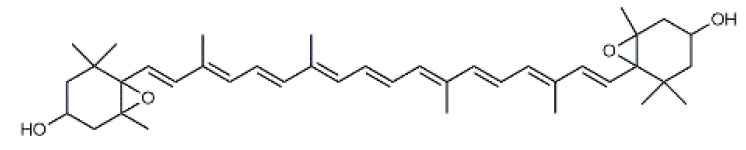 C_40_H_56_O_4_ M = 600.88 g·mol^−1^	(+) Catechin (**11**) and epicatechin differentiate in stereochemistry of C2 OH-gr 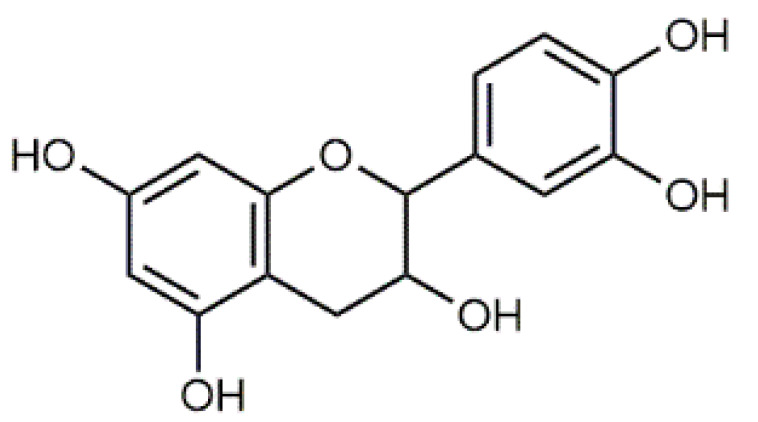 C_15_H_14_O_6_ M = 290.27 g·mol^−1^	Dermorubin (**15**) 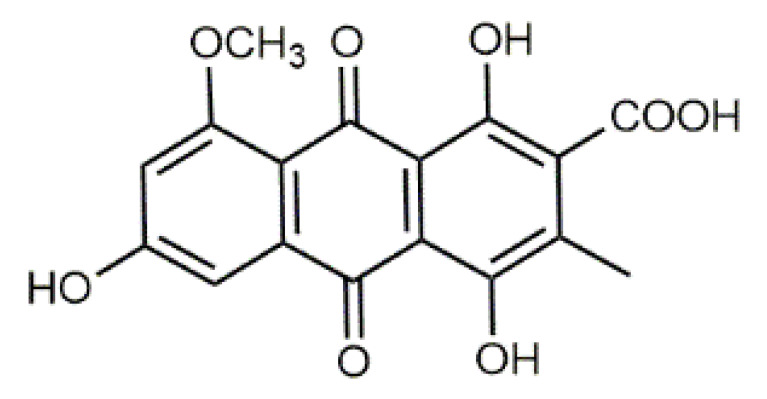 C_17_H_12_O_8_ M = 344.28 g·mol^−1^	Leucomentin 3 (**19**) 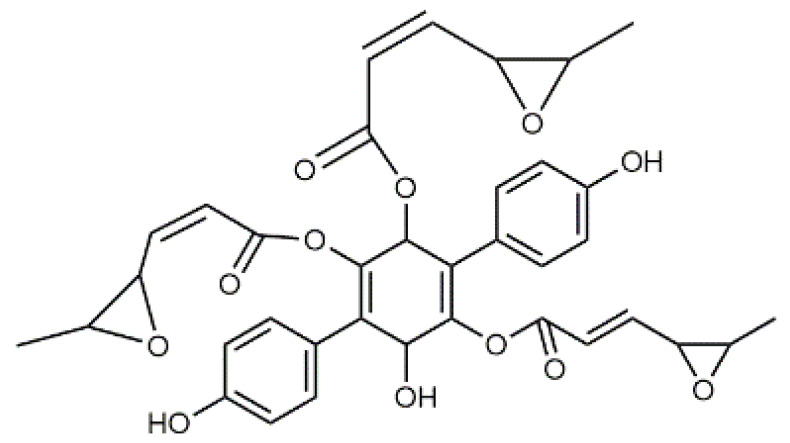 C_36_H_34_O_12_ M = 658.66 g·mol^−1^
	β-Carotene (**8**) 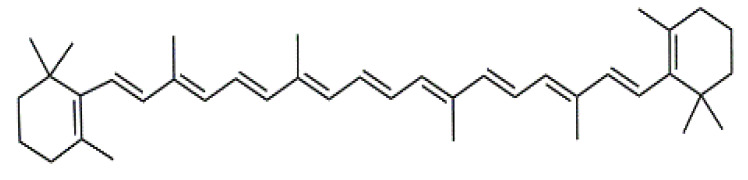 C_40_H_56_ M = 536.89 g·mol^−1^	Salireposide (**12**) 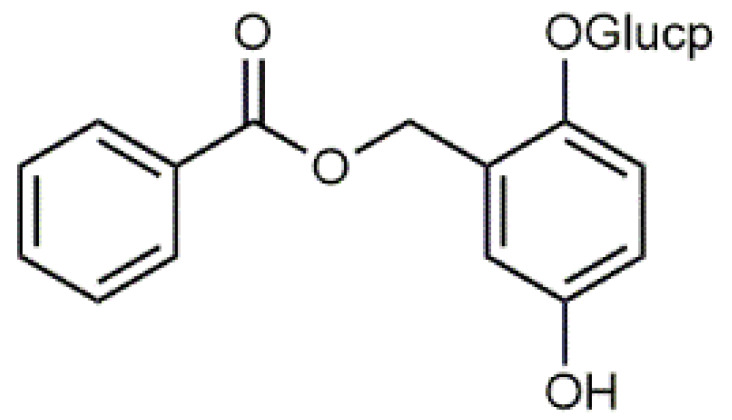 C_20_H_22_O_9_ M = 406.37 g·mol^−1^	Physcion (**16**) 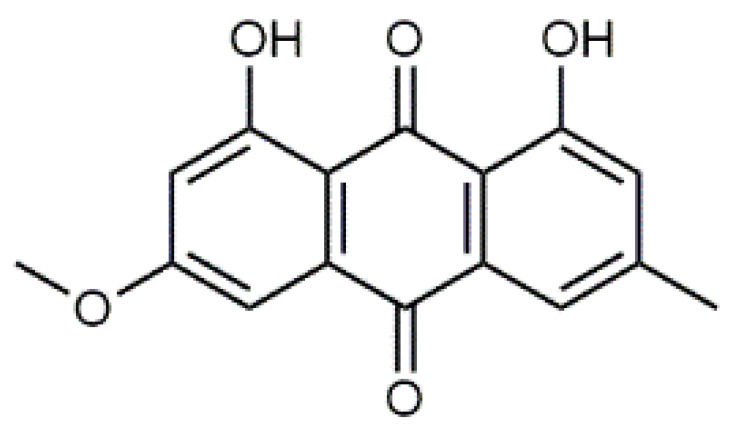 C_16_H_12_O_5_ M = 284.27 g·mol^−1^	Spiromentin (**20**) 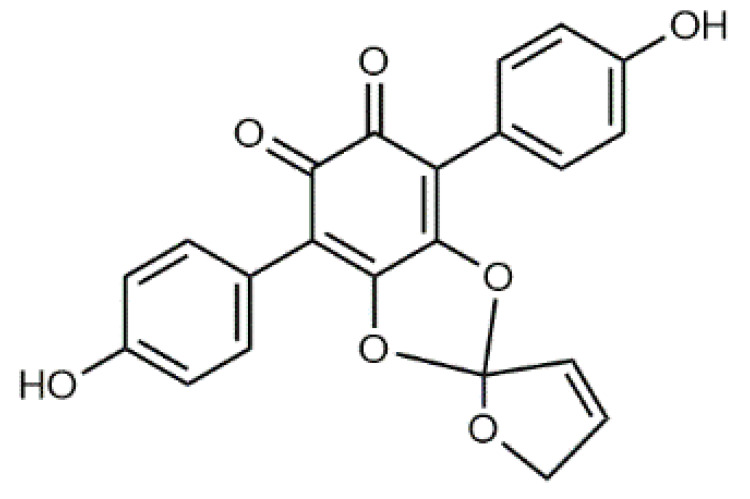 C_22_H_14_O_7_ M = 390.35 g·mol^−1^

**Table 3:** The results of the HPLC-UV/Vis-MS data of *Tanacetum vulgare*, *Cortinarius semisanguineus* and *Tapinella atrotomentosa*. The chemical structures and the calculated MS of the numbered compounds are found in Table 2. The MS data for *C. semisanguineus* refers to our earlier data published in [68] and [85].

Ret. Time	UV/Vis λ_max_ [nm]	MS Data: *m/z* [M − H]/[M + H]^+^ and Main Fragment	Molecular Formula	Compound
*Tanacetum vulgare*, Figure 1, chromatogram (370 nm) and spectra A				
13.61	218, 244, 298 sh ^‡^, 328	353.0864; 191.0535	C_16_H_18_O_9_	Caffeoylquinic acid isomer I
25.42	204, 256, 366	463.0878; 301.0331	C_21_H_20_O_12_	Quercetin (**6**) derivative
27.10	218, 244, 298 sh, 328	515.1186; 353.0859	C_25_H_24_O_12_	Caffeoylquinic acid isomer II
29.75	200, 266, 336	445.0759; 269.0428	C_21_H_18_O_11_	Apigenin derivative
30.13	218, 244, 300 sh, 328	515.1191; 353.0863	C_25_H_24_O_12_	Caffeoylquinic acid isomer III
31.55	204, 254, 266, 346	639.3185; 285.0378	-	Luteolin (**5**) derivative
40.09	206, 266, 336	269.0427	C_15_H_10_O_5_	Apigenin
41.13	204, 254, 266, 346	285.0388	C_15_H_10_O_6_	Luteolin (**5**)
*Cortinarius semisanguineus*, Figure 1, chromatogram (440 nm) and spectra B				
15.91	224, 286, 438	327; 283	C_17_H_12_O_7_	Dermolutein
16.64	250, 285, 434	-	C_17_H_11_ClO_7_	5-Cl-Demolutein
18.16	224, 288, 439	684	C_21_H_20_O_10_	Emodin-1-glucoside (**13**) *
18.86	231, 278, 482	343; 299	C_17_H_12_O_8_	Dermorubin (**15**)
20.23	229, 285, 452	303; 239	C_15_H_9_ClO_5_	7-Cl-Emodin
23.03	211, 283, 431	283; 240	C_16_H_12_O_5_	Physcion (**16**)
26.28	263, 484	770	C_22_H_22_O_12_	Dermocybin-1-glucoside (**14**) ^#^
*Tapinella atrotomentosa*, Figure 1, chromatogram (370 nm) and spectra C				
07.40	263, 371	325.07; 307.06	C_18_H_12_O_6_	Atromentin (**17**)

^‡^ shoulder, * Emodin-1-glucoside heksaacetate, ^#^ Dermocybin-1-glucoside heptaacetate.

**Table 4:** Colour fastnesses to light (scale 1−8) and washing (scale 1−5). LF = light fastness (200 h without side change), Cc = colour change, s = staining, NM = no mordant.

Dyeing	LF	Washing Fastness		
		Cc	s, WO	s, CO
*Tanacetum*				
NM, direct dye	6	1	5	4/5
Alum mordant	7	3/4	5	4
FeSO4 mordant	7	2/3	5	4
*Salix*				
NM, direct dye	5	1	5	5
Alum mordant	6	2/3	5	5
FeSO4 mordant	6	2	5	5
*Cortinarius*				
NM, direct dye	3	3/4	4/5	4/5
Alum mordant	4	4	4/5	4/5
FeSO4 mordant	5	4	4/5	4/5
*Tapinella*				
NM, direct dye	2	3	5	4/5
Alum mordant	4	3/4	5	4/5
FeSO_4_ mordant	5	3/4	5	5
